# My Home is my Castle? The Role of Living Arrangements on Experiencing the COVID-19 Pandemic: Evidence From Germany

**DOI:** 10.3389/fsoc.2021.785201

**Published:** 2022-01-11

**Authors:** Alexander Langenkamp, Tomás Cano, Christian S. Czymara

**Affiliations:** ^1^ Goethe-Universitat Frankfurt am Main, Sociology, Frankfurt am Main, Germany; ^2^ Universidad Nacional de Educación a Distancia (UNED), Madrid, Spain

**Keywords:** loneliness, crisis, social inequality, living conditions, well-being, COVID-19, household structure

## Abstract

During the early months of the COVID-19 pandemic in Germany, social restrictions and social distancing policies forced large parts of social life to take place within the household. However, comparatively little is known about how private living situations shaped individuals experiences of this crisis. To investigate this issue, we analyze how experiences and concerns vary across living arrangements along two dimensions that may be associated with social disadvantage: loneliness and care. In doing so, we employ quantitative text analysis on open-ended questions from survey data on a sample of 1,073 individuals living in Germany. We focus our analyses on four different household structures: living alone, shared living without children, living with a partner and children, and single parents. We find that single parents (who are primarily single mothers) are at high risk of experiencing care-related worries, particularly regarding their financial situation, while individuals living alone are most likely to report feelings of loneliness. Those individuals living in shared houses, with or without children, had the lowest risk of experiencing both loneliness and care-related worries. These findings illustrate that the living situation at home substantially impacts how individuals experienced and coped with the pandemic situation during the first wave of the pandemic.

## Introduction

At the outset of the COVID-19 crisis in Germany, living arrangements and household structure suddenly became of critical importance for individuals’ wellbeing due to stay-at-home orders. But some types of living arrangements or household structures might be less beneficial than others in helping individuals to cope with the negative effects of the pandemic. For example, those living alone or heading a single-parent family have been found to be at particular risk of social disadvantage, both before and during the COVID-19 pandemic (Burström and Tao, 2020; Gray et al., 2020). Therefore, documenting variations in how individuals have experienced the COVID-19 pandemic across different types of living arrangements provides new insights into how household structure influences individual experiences in times of crisis.

Studies on the social and economic impacts of the pandemic and the related countermeasures highlight mostly negative effects for most individuals and families across a wide range of outcomes: declines in mental health (Pfefferbaum and North, 2020), increases in unemployment (Bauer and Weber, 2020), rising inequality in time devoted to childcare and housework (Kreyenfeld et al., 2020; Möhring et al., 2020) and cultural capital (Jæger and Blaabæk, 2020), reduced social trust and wellbeing (Sibley et al., 2020), or unequal cognitive labor of unpaid work (Czymara et al., 2020). We contribute to this growing body of literature on COVID-19 and social inequality by exploring variations in experiences based on living arrangements, focusing on Germany as a case study. Given the measures taken to control the spread of the virus, e.g., stay-at-home orders and social isolation, studying how household structures and living arrangements are associated with different types of experiences is of particular interest. This is because understanding how the restrictions’ negative effects are unequally distributed across living arrangements is essential to informing policy about who faces greater risks of psychological distress as a result of these types of measures. Correspondingly, the objective of this exploratory study is to investigate how individuals experienced the first wave of the coronavirus pandemic across living arrangements in Germany.

The analysis is based on an online survey fielded at the beginning of the pandemic in Germany (n = 1,073). In this survey, we collected socio-demographic information together with an open question that asked participants to describe how they experienced the COVID-19 pandemic in a narrative form. To analyze these narratives, we employ quantitative network analysis of words and probabilistic topic models. The four types of living arrangement we focus our analyses on are as follows: 1) single households, 2) parents living with children, 3) single parents, and 4) individuals living with others, but without children, hereafter referred to as *shared living without children*.

The study is structured in four parts. First, we describe the situation in Germany in March and April 2020 and the implications of the pandemic for individuals living in the four considered household types. We do so to provide the reader with more contextual information about the case study and the investigated living arrangements. Secondly, we present the data and methods used in the empirical assessment. Third, we present the results of the analyses conducted on the data collected from the online survey. Finally, the discussion section summarizes the key insights, limitations, and how the study speaks to the current debate about how the pandemic relates to household structure, living arrangements, and social inequality.

## COVID-19, Social Distancing and the New Role of the Household

In March 2020, rising numbers of COVID-19 infections were registered in Germany for the first time. As little was known about the virus at this time, Germany introduced a variety of policies to slow the spread of the virus. These included, among others, the closure of restaurants, schools, and childcare facilities, the prohibition of public events, and restrictions on gatherings of more than two people from different households (Bennhold and Eddy, 2020; Bundesgesetzesblatt, 2020; Donsimoni et al., 2020). Taken together, these restrictions were significant interventions in public and private life. For instance, these restrictions effectively forced a large part of the labor force to relocate their working space to their own home (Möhring et al., 2020). Likewise, the closed schools, childcare facilities and contact restrictions challenged working parents to combine private life, work, and childcare in the same space, often without the possibility of receiving help from individuals not living in the same household. In addition to challenges related to harmonizing those spheres of life, many households faced issues due to the economic shock of the pandemic and related job losses (Nitt-Drießelmann et al., 2020; Möhring et al., 2021).

The respondents’ experiences during this time are likely characterised by changes in their social lives, financial situations, and conflicts at home. However, given the central role of the household during the restrictions, we expect the type of living arrangement to be a key factor in explaining variations in concerns. Namely, the closure of childcare facilities and the restrictions of social life likely play a major role in individual experiences of the crisis. Thus, we expect that two aspects in particular are core drivers of differences in worries across groups:

We distinguish groups along two corresponding dimensions: whether they have direct responsibility for children, and whether they have a higher risk of loneliness due to their living arrangements. Table 1 provides an overview of this conceptualization. Our analysis differentiates between four groups: 1) those *living alone*, who have less or no responsibility for children but a higher risk of loneliness (upper left cell of Table 1); 2); *single parents*, i.e., individuals who have at least one child they have to take care of but who do not live with a partner (upper right cell of Table 1); 3) *couples with* (a) *child(ren)*, often regarded as the “standard family” (lower left cell of Table 1); and 4) those who *live together with another person* (e.g., partner or roommate) but *do not have children* (lower right cell of Table 1). We focus on these four types of living arrangements for two reasons. First, they have the highest prevalence in contemporary Germany. Second, they provide different family configuration types. Each of them provides distinct social, emotional and economic resources to compensate for the plausible effects of the pandemic on individuals’ mental and physical wellbeing. Next, we review each of the four categories in detail to provide more context for the subsequent analysis.

**TABLE 1:** Types of living arrangements.

		Responsibility for children	
		Lower	Higher
**Risk of loneliness**	**Higher**	Living alone	Single parents
	**Lower**	Shared living without children	Couples with child(ren)

First, we discuss single households without children. After the social distancing policies were put in place, single household occupants were severely limited in their ability to meet family and friends. Although epidemiologically necessary, social distancing policies contradict the human need for social interaction and intimate social encounters (Baumeister and Leary, 1995; Bavel et al., 2020). Social connections are an important emotional resource and foster emotional stability, stress resilience, and general wellbeing. In line with that, decades of research highlights that deprivation of social interaction has severe consequences for physical and mental health, including diminished sleep quality, depression, anxiety or even cardiovascular illnesses (Hawkley and Cacioppo, 2010; Holt-Lunstad et al., 2015). Consequentially, people are typically reluctant to renounce their social interactions, and studies show a surge in digital communication as well as an increase in stress symptoms during quarantine (Cellini et al., 2020). Likewise, research found increased prevalence of depression and anxiety during lockdown in Germany (Benke et al., 2020).

Given that they are living alone, digital communication became especially important for single household occupants. However, research suggests that digital communication only partially compensates for the loss of face-to-face contacts (Helliwell and Huang, 2013; Verduyn et al., 2015). As digital communication seems insufficient to fully compensate for lack of face-to-face contact, it can be expected that individuals living alone suffer from social isolation and loneliness in particular, which is associated with a decline in mental health and an increase in symptoms of depression. Taken together, the laws that limited public gatherings to only two individuals from separate households as well as the closure of public places and events burdened single household occupants in particular.

Second, we consider individuals who live with other adults and without children. This group includes a diverse range of living arrangements such as romantic couples, roommates, or intergenerational living arrangements. All households in this group have in common that they had the opportunity to socialize with their cohabitants during the period of restricted opportunities to gather outside one’s own household. Importantly, these households also did not suffer from the closure child care facilities. While care arrangements for people of advanced age are present in this group as well, medically justified care support was not restricted during this time in Germany.

There are two noteworthy effects of cohabitation: first, cohabitation can have several positive effects. Research has shown that the first three supportive social contacts play an important role in shielding a person from loneliness (Van Tilburg, 1990); additional relationships have only diminishing benefits. Furthermore, psychological studies show that these individuals may be less psychologically affected by the COVID-19 restrictions as social relationships benefit interpersonal emotional regulation (Zaki and Craig Williams, 2013; Williams et al., 2018). Therefore, while those living alone during the COVID-19 pandemic might be at particular risk of suffering from mental health issues linked to loneliness, shared living likely protects individuals from loneliness during times of social distancing policies. Not only can cohabitants protect each other from isolation through more emotional support, in some cases they can also compensate for each other in the event of income loss or unemployment spells. Indeed, research has shown that intimate partnerships especially exert a positive influence on the ability to cope with personal crises such as financial difficulties, war, or illness (Pinkerton and Dolan, 2007; Benzies and Mychasiuk, 2009; Figley and McCubbin, 2016). In addition, cohabiting couples without children are able to avoid a particularly critical source of stress during the COVID-19 pandemic: increased levels of childcare needs due to school closures.

However, shared living can also raise stress levels, which in some cases may ultimately lead to violence, a phenomenon documented in the context of romantic relationships and families in particular (Schneider et al., 2016; Kofman and Garfin, 2020; Mazza et al., 2020). For instance, social scientists have long studied the relationship between macro-economic conditions and intimate partner violence (a classic study is Elder, 2019). Because intermittent income, unemployment, stress, and to some extent isolation are associated with an increased risk of domestic violence, cohabiting childless couples may also experience more conflict during the COVID-19 crisis (Capaldi et al., 2012; Lucero et al., 2016). Being “trapped” together at home without many outdoor activities may also be fruitful ground for conflict, abuse, and violence. In fact, several studies have already shown an increase in domestic violence during lockdown conditions (Simonovic, 2020; Zhang, 2020). Therefore, it can be expected that the topics covered in narratives of individuals in cohabitation without children are more diverse and less focused on social isolation but rather on the positive as well as the negative aspects of cohabitation itself.

The third group we analyze is the “standard” family, i.e., married or cohabiting couples with children. This type of living arrangement primarily differs from the previous ones in that there are children at home. In light of the restrictions on child care facilities, households with children were increasingly burdened with childcare needs and unpaid labour during the pandemic. Likewise, the increased necessity to work from home challenged the parents to harmonize paid labour and private life. This issue became amplified when external support for childcare and housekeeping were prohibited due to the contact restrictions and closures of schools and daycare centers, which led to a massive increase in unpaid household labor (both childcare and housework) during that time.

Therefore, cohabitation with children during the pandemic entails unprecedented complications and difficulties for caretakers. In addition, families, especially children and mothers, faced new challenges with limited outdoor activities and the lack of play with friends and peers (Huebener et al., 2020; Zinn and Bayer, 2020). Likewise, children have experienced significant learning losses during school closures, while parents devoted much more time and effort to the aforementioned unpaid work (Andrew et al., 2020; Czymara et al., 2020).

However, having more time for family during lockdown and providing each other with company in times of crisis can be expected to be part of the experience of this group as well. In respect to finances, household stability and the “standard” family structure have a positive association with socio-economic status: higher income and better-educated individuals are more likely to live in this type of arrangement (Esping-Andersen and Billari, 2015; Esping-Andersen, 2016). While many people, independent of household type, will experience at least concerns about their financial situation in near future, we expect this topic to be present but not of immediate concern for these so-called standard families, as they have more resources to compensate for the socio-economic shock ushered in by the pandemic. Thus, we would expect the narratives of cohabiting parents to cover worries and concerns related to childcare, work, household conflict, and some financial issues.

In light of recent findings indicating that marriage has a very similar effect on subjective wellbeing as cohabitation (Perelli-Harris et al., 2019), we do not further differentiate between both groups. All challenging aspects of the pandemic mentioned thus far, e.g., childcare, financial concerns, restrictions on social life, and loneliness, are amplified for single parents who have to combine work and childcare without the support of a partner. This is the fourth group of our analysis. While couples are potentially able to partially offset a partner’s lost earnings, single parents can face severe financial problems as the sole provider of their household. In addition, single parents may see their time squeezed to its limits, as they face a double inability to distribute unpaid work due to COVID-19 restrictions: they neither have a partner to contribute to necessary but unpaid work, nor can they leave children with other relatives (mainly grandparents, normally). Because time is constrained, single parents face important obstacles in combining employment and care. Furthermore, roughly 90 percent of single-parent families in Germany are female, a group that already held a disadvantaged position in the labor market before the outbreak of the pandemic (BIB, 2020; Dernberger and Pepin, 2020). Single-mothers’ disadvantages in the labor market compared to the time before the outbreak of COVID-19 seem to have widened, as the current crisis is impacting feminized occupations more than masculinized ones (Alon et al., 2020). Therefore, we expect narratives of single parents to be dominated by reports of financial struggles, issues related to childcare, and general distress.

## Data and Methods

We draw upon an online survey fielded in Germany between March 27 and April 26, 2020, which were the first weeks of the COVID-19-related restrictions, to examine personal experiences with these policies for different living situations. The data are available at: https://doi.org/10.7802/2034; the underlying code is available at: (https://dx.doi.org/10.17605/OSF.IO/MVA5W). We created the online questionnaire using SoSciSurvey (Leiner, 2020). The survey was advertised in a press release by Goethe University Frankfurt and was shared on the *Psychologie Heute* website and on the official Facebook pages of German cities^1^. Participation in the survey was voluntary and no financial incentive was given. After a set of close-ended questions, the participants were asked to answer an open-ended question about their personal experience with the COVID-19-related restrictions. This item was introduced with the prompt: “The social lives of many people have changed as the coronavirus spreads. Here you get the opportunity to report your personal situation, your family life or your experience of being together in general.” The question was neutrally worded to avoid priming individuals to report only positive or negative narratives, and it does not hint at our expected topics. In total, 2,274 individuals participated in the survey. Of those, 1,119 participants provided answers to the open-ended question. After removing short, and thus unusable, answers, our analysis is based on 1,073 respondents. While couples with children wrote the shortest answers (mean: 533.66), those living alone wrote with an average length of 657.76 characters (see Table 2). For our analysis, we removed stop words and non-character symbols from these answers, and stemmed words using Silge and Robinson (2016) and Benoit et al. (2018). For topic models, we also removed words that occurred in more than 20 or less than 0.1 percent of all answers to reduce dimensionality.

**TABLE 2:** Descriptive statistics.

Variable	N	Single parents (n = 53)	Couples with child(ren) (n = 328)	Living alone (n = 199)	Shared living without children (n = 493)
Length of response (in characters)	1,073	623.60	533.66	657.76	526.65
Gender					
Men	224	9.43%	19.10%	19.82%	23.63%
Woman	841	90.57%	80.04%	79.88%	75.56%
Other	6	0.0%	0.5%	0.3%	0.81%
Age					
0–17	5	1.89%	0%	0.51%	0.61%
18–29	240	5.66%	3.38%	21.83%	37.5%
30–44	351	28.3%	55.38%	23.35%	22.54%
45–59	320	56.6%	37.23%	30.96%	22.13%
60–74	147	7.55%	4%	23.35%	17.21%
Education					
Primary level or less	22	1.89%	0.92%	2.04%	2.87%
Secondary level	355	43.4%	31.08%	31.12%	34.84%
Tertiary level	685	54.72%	68%	66.84%	62.3%
Financial satisfaction					
Not satisfied	30	3.85%	1.55%	3.13%	3.5%
Rather unsatisfied	132	17.31%	9.01%	20.31%	11.32%
Rather satisfied	605	65.38%	58.39%	54.69%	57.2%
Satisfied	285	31.46	31.06%	21.88%	27.98%

We are interested in differences in worries based on peoples’ living arrangements. Our survey asked respondents, “Which of the following best fits your living situation?”, with six possible answers: “Living with partner and children” (hereafter: couples with child(ren)); “Living with partner”; “Living alone”; “Living without partner, but with children” (hereafter: single parents); “Living in another kind of shared apartment”; “None of the above options”. Our analysis does not include those in the last category (52 observations) or those with missing values (3 observations). In line with our conceptual model shown in Table 1, we combined couples without children and those who share their apartment in another way into the category, Shared living without children. Table 2 breaks down the descriptive statistics for each of the four groups^2^.

It is important to note that the convenience sample, similar to most explorative studies, is not representative for the German general population. As Table 2 shows, our survey oversampled women with higher education. In total, about 80 percent of our final sample were women: 70 percent were between 30 and 59 years old and almost two thirds had completed tertiary education. However, this was not a problem because our main interest lies in comparing different types of living arrangements. Important for the comparison of narratives by household type, the distributions between the four types of living arrangements are mostly similar, with roughly 75 to 80 percent being women, and 62 to 68 percent having tertiary education. That an even greater share of single parents are women (over 90 percent) is in line with findings from previous research, as women usually take care of children after partnership dissolution in Germany and other Western countries (Geisler and Kreyenfeld, 2019; Cano and Gracia, 2020). The fact that basic sociodemographics distribute largely similarly across living arrangements is beneficial for our analysis because we do not have to apply weights to account for different sociodemographic compositions between household types. However, this study likely underreports e.g., financial struggles, which correlate with socio-economic status. Moreover, note that the number of observations for each group differs, but that it is large enough for meaningful results in every case. The number of observations ranges from 53 for single parents to 493 for people not living alone but living with children (see Table 1).

### Analytical Strategies

Our quantitative content analysis of the responses to the open-ended survey question is based on two approaches: Word correlation networks and topic models. These tools allow us to identify the most common topics and word configurations present in the narratives. Subsequently, we illustrate important topics reported by household type using quotes form the narratives.

### Word Networks

**FIGURE 1:**
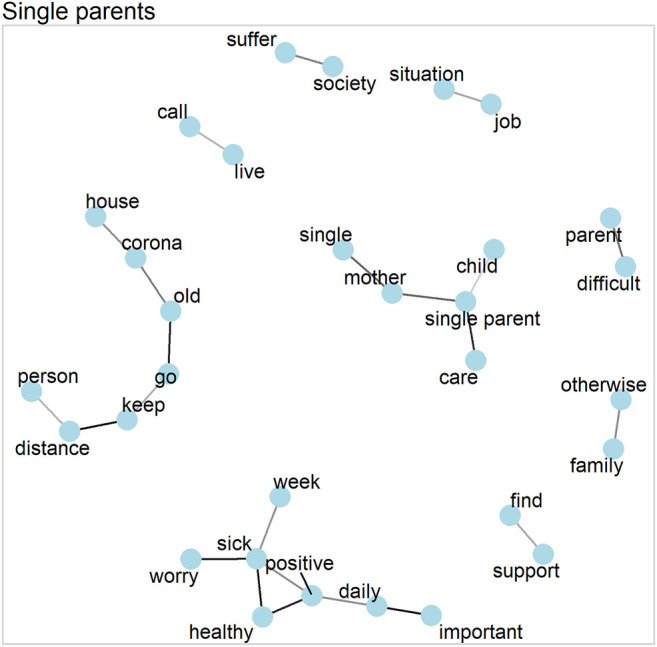
Word network for single parents (correlations >0.5).

Word networks yield information about the connection of narratives by showing how word clusters relate to one another. They are based on the correlations of words within each household. To this end, we calculated the pairwise correlations of words that occurred at least 20 times in total. Below we show networks of words based on these pairwise correlations (Silge and Robinson, 2017). Figures 1,3,5,7 show the correlations between words, with each line indicating a correlation larger than 0.25 (and larger than 0.5 for single parents) and darker lines indicating stronger correlations (i.e., no line represents a correlation below 0.25 or 0.5 respectively).

### Topic Models

We are also interested in the salience of different topics in the four household groups. Topic models are ideally suited for this purpose (Roberts et al., 2014; Boumans and Trilling, 2016). We use the structural topic model (*stm*) package of Roberts et al. (2014), which defines topics as words that are more probable to appear together in a given text (Boumans and Trilling, 2016). STM calculates probability models to estimate how a pre-defined number of topics (in this study, eight) distribute across the data. This results in two posterior probability distributions: the distribution of terms within each topic (1) and the distribution of topics within each survey answer (2). The former (1) indicates the likelihood that each term appears in a certain topic, theoretically ranging from zero (certainly does not belong to this topic) to one (certainly belongs to this topic). The latter (2) shows the probabilities of each topic in a given survey answer article, also potentially ranging from zero (does not appear in this answer at all) to one (this answer consists only of this topic). The probabilities of all terms to belong to a survey answer add up to a sum of one and so do the probabilities of all topics to belong to an article (Roberts et al., 2014; Boumans and Trilling, 2016). The data were cleaned and analyzed separately for each group to get a clearer picture of how narratives generally differ depending on one’s living arrangement.

### Content Analysis

Our data offer a rich qualitative component in form of narratives. We take advantage of these data to show exemplary answers of relevant topics to illuminate the topics’ content, separating by type of living arrangement. These answers are taken from the most probable answers for each topic, defined by the second probability distribution mentioned above. In other words, the answers we show below were the most representative of each topic. This type of analysis allows us to describe in a deeper way the most representative experiences associated with each of the four analyzed household structures.

## Results

### Single Parents

**FIGURE 2:**
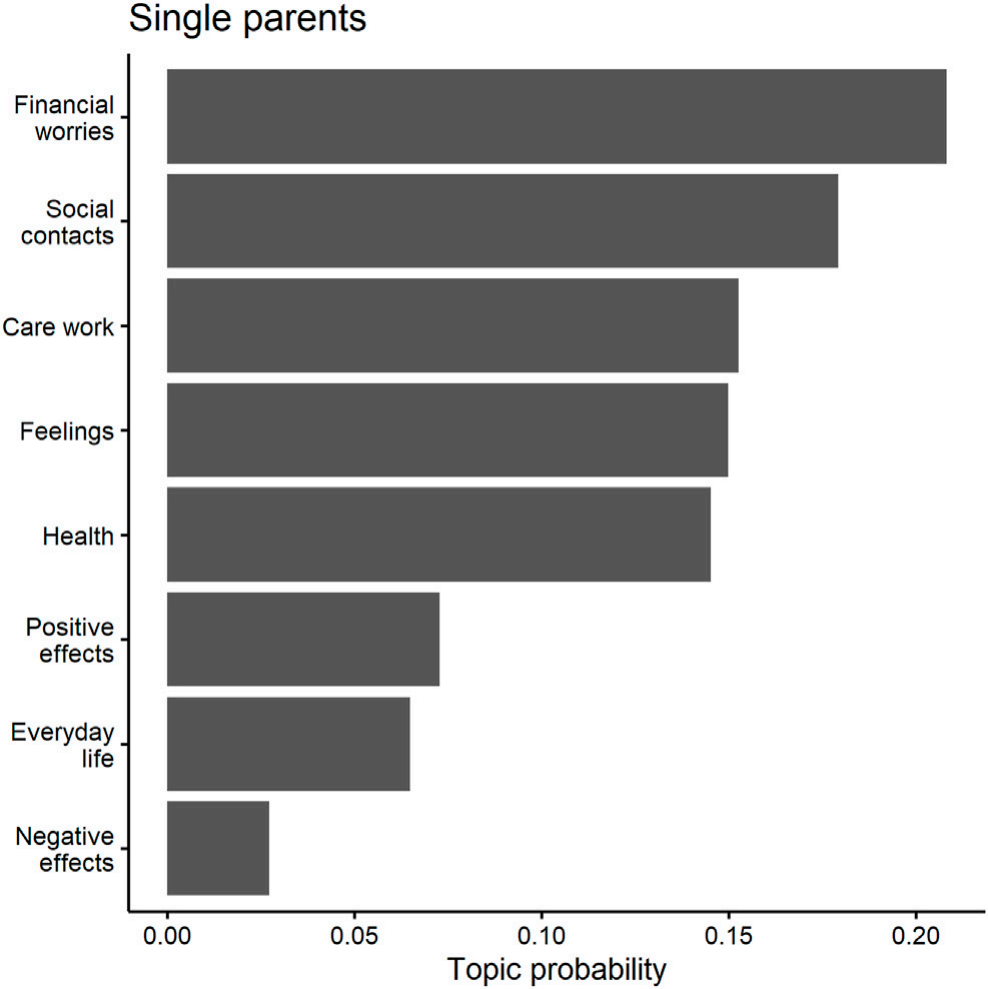
Predicted topic probabilities for single parents.

The word correlations in Figure 1 show that single parents—which mainly means *single mothers* here—have several separate thematic clusters. These clusters relate to caring for a child and worrying about health or employment. The results of the topic models, which are shown in Table 3, confirm the importance of these issues for single parents. Most importantly, the fourth topic of the model clearly relates to financial struggles with terms such as *restrict*, *financial*, *worries*, and *difficulty*. As Figure 2 shows, this topic dominates the single parent narratives, with a probability of 23.2 percent. Noticeably, none of the other three living arrangement groups has one topic that stands out this strongly (see below).

**TABLE 3:** Topics of single parents (n = 53).

Topic 1	Topic 2	Topic 3	Topic 4	Topic 5	Topic 6	Topic 7	Topic 8
live	care	burden	restrict	feel	live	find	together
enjoy	special	feel	financial	hope	week	school	shopping
positive	entertain	suffer	since	find	healthy	shopping	phone
see	look after	people	son	fast	son	difficult	meet
important	get	job	worries	see	sick	help	keep
possible	difficult	grown-up	difficult	office	mask	alone	missing
air	grown-up	crisis	healthy	home	important	climb	part

The most probable answer for the fourth topic illuminates its content. The answer with the highest topic probability comes from a single mother who is between 45 and 59 years old and has a medium level of education (i.e. secondary level). She feared an existential crisis due to the COVID-19 restrictions, relating to her life as well as the lives of those for whom she cares:

“I work full time. I recently lost my second 450 Euro job because the (…) is closed. Now I am scared that I won’t be able to keep my house without this side job. That would be a disaster. And I am really scared about the future because my son-in-law and my son could also become unemployed thanks to the Corona crisis.

The longer it lasts, the sooner I will lose my house, and my children won’t be able to build a future. Shocking. And I am very afraid for my old parents (91 and 85 years). We can only talk when one of us is on the balcony and the other is in the courtyard. It’s absolutely terrible for all of us.”^3^

Single parents were also concerned about a lack of social interactions, as indicated by the eighth topic in Table 3, which consists of terms such as *together*, *meet*, *missing*. This topic was the second most prevalent topic for single parents, with a share of 14.9 percent (Figure 2). Single parents seem to struggle with lack of social contact, as the two comments with the highest probability for this topic indicate. The answers below come from two mothers who are both between 30 and 44 years old and have completed higher education. The first single mother was a full-time employee and stated that:

“I cannot leave the house together with my children and partner, who does not live with us … For single parents, this 24 h care work for weeks on end is an extreme burden, because all of us (single parents) lack a social life.”

The second single mother, who was employed part-time at the time of the survey, says:

“As a single mom, my social life, even before the Corona crisis, consisted mainly of my contacts at work, daycare and family life (parents and sister’s family). Meeting friends, going to events, or traveling was rarely possible for me even before.”

However, single parents were not only worried about their own social lives: they were also concerned about their children’s lack of social interaction, as the following answer from a single mother of the same age range makes clear:

“As a single parent, working from home with a child is exhausting. My child misses playing with other children, which is really important.”

Some respondents tried to keep a more positive perspective, substituting the missing personal contact with phone calls or online chats. For example, one single mother, who was between 45 and 59 years old, said:

“I miss personal contact, but there is always the telephone. Good friends are the best psychologists and are always there.”

The third most salient topic for single parents is the third topic of Table 3, which consists of terms such as *burden*, *suffer*, and *crisis*, indicating that the situation indeed hit single parents hard. It reflects the combination of childcare and work-related stressors and occurs in the single parent group with a probability of 14.2 percent. Topic 3 is illuminated by the answer of one single mother, who summarizes her worries in the following way:

“Social isolation is incredibly stressful, especially for my child. If the schools stay closed long-term, we’ll have even more intra-family conflicts. Homeschooling and a full-time job is a double burden that is very difficult to cope with under these circumstances. The social ‘uncoupling’ within my own circle of friends is causing me to have strong mood swings and emotional stress.”

These worries are shared by a student in her twenties, also a single mother, who reports:

“The current situation is very stressful. My son cannot go to kindergarten and he is often in a bad mood and bored. This stresses me out and I only feel irritated.”

These results show the clear dominance of financial worries and lack of social interaction for single parents. This is in line with our theoretical expectations, that the responsibility for children in combination with a high risk of loneliness (see Table 1) made single parents a particularly vulnerable group during the pandemic related lockdowns.

### Couples With Child(ren)

**FIGURE 3:**
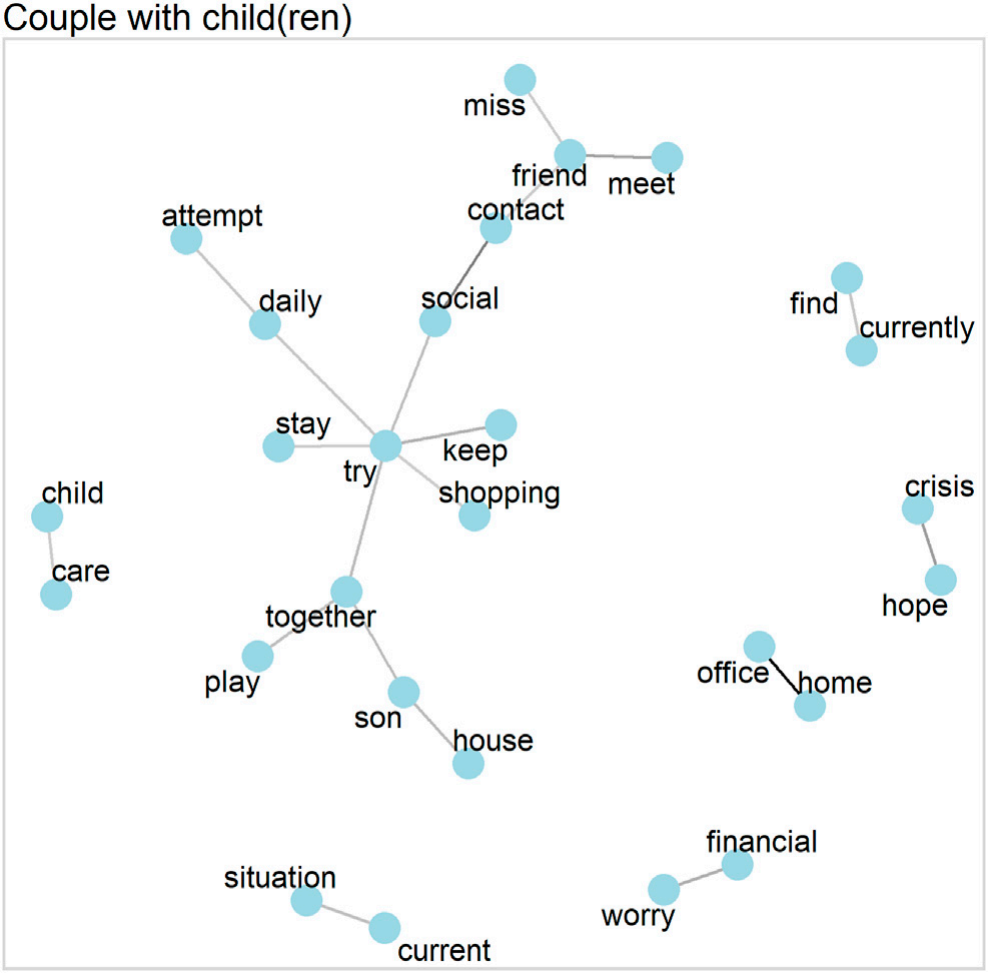
Word network for couples with child(ren) (correlations >0.25).

In contrast to single parents, households in which at least one child lives together with both parents (i.e., couples with children) have one larger word cluster, as Figure 3 shows. This cluster corresponds to organizing everyday life with a child (playing together, going shopping, and meeting as well as missing friends). However, they also reported financial worries and about problems related to working from home.

**FIGURE 4:**
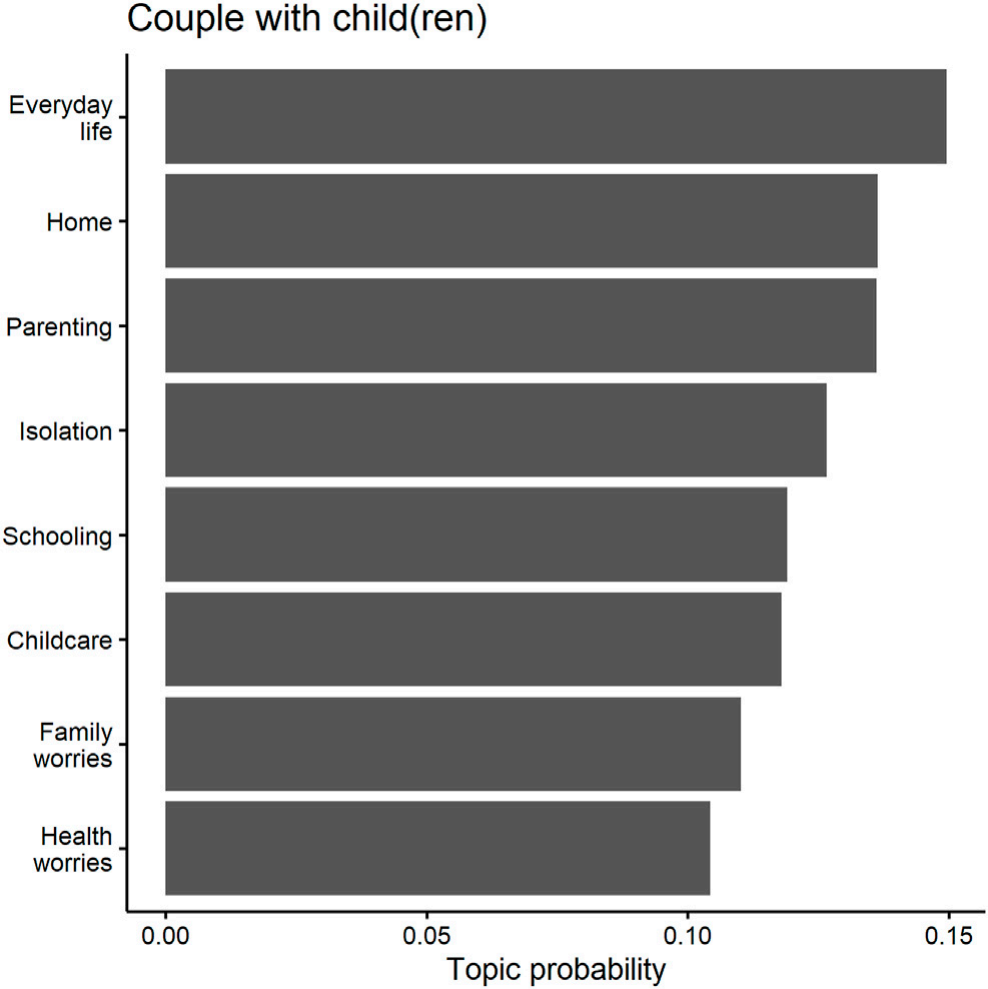
Predicted topic probabilities for couples with child(ren).

The topic models, shown in Table 4 and Figure 4, confirm that couples with child(ren) mostly addressed childcare and family-related issues. In contrast to single parents, the distribution of topics for couples with child(ren) is more equal, with every topic occurring with a probability of around 10 to 15 percent, while the three most prevalent topics (topics 3, 4 and 5) all have a probability of about 15 percent (see Figure 4). Topic 4 of Table 4 relates more to the general handling of family-related duties, including negative (*crisis*, *worry*, *burden*) as well as more neutral to positive (*together*, *spending time*) aspects of childcare. Examining the answers with the highest probabilities of this topic tends to show a rather positive picture within this group. One mother, aged 30 to 44 and with a university degree, replied:

“The crisis became an opportunity to restructure family life. My husband’s employer expanded their work from home options so that we could set up a joint plan to care for of our children together. Now that the kids aren’t going to school, which forced us to wake up early in the morning, we have been able to find our own daily routine. The children sleep in and eat breakfast in peace and quiet before starting on their homework. We can support them and answer their questions, while they get a new perspective on their assignments. We eat every meal together, talk and discuss things in peace. In the evening, the children are allowed to play together for a while (according to their rhythm). This has brought us closer together as a family because many external factors have disappeared and we cannot escape from real life by going on vacation. We personally would welcome working from home on a permanent basis.”

**TABLE 4:** Topics of couples with child(ren) (n = 328).

Topic 1	Topic 2	Topic 3	Topic 4	Topic 5	Topic 6	Topic 7	Topic 8
important	house	house	together	worry	see	strong	school
current	parents	person	crisis	shopping	home	crisis	find
daughter	social	home office	person	parents	corona	worry	house
house	suffer	garden	worry	missing	family member	home office	try
high-risk group	we	enjoy	childcare	home office	alone	changed	possible
school	keep	meet	spend time	find	social	everyday life	keep
hope	shopping	week	burden	do	land	evening	visit

A father from the same age group reports a similar situation:

“Losing my job resulted, so to speak, in a kind of “forced vacation,” so to speak, which has been positive, though. I have more time for family (i.e. wife and child), housework, gardening, relaxation, walks, and for visiting one family friend (exclusive arrangement to meet privately only with one another and otherwise to keep the contact ban).”

A functional family might even increase resilience in facing the personal and social consequences of the pandemic-related restrictions. A self-employed father with a higher education, aged 30 to 44, reported:

“Living with the family (my partner and her son) has a stabilizing function. I don’t notice any loneliness or isolation in everyday life. Rather, in this close circle, all of the demands can be stressful and there is little time left for oneself. Nevertheless: we support one another and brighten up each other’s days.”

However, the situation was not exclusively positive for couples with children. This is shown by topic five in Table 4, which deals with family-related concerns, indicated by terms like *worry*, *parents*, or *missing*. An exemplary response from this topic was from a mother in the 45 to 59 age range, who was employed part-time and has a university degree and a child in adolescence. She wrote:

“It is an emotional challenge sitting stuck in one’s own home and working, with a teenager, who is being digitally educated for the first time and who has to be forced to get fresh air, and simultaneously worrying about my old and sometimes very sick parents. I’m almost certain that my 88-year-old father with dementia will not recognize me if I’m allowed to visit him in the nursing home again at some point. Apart from work and emotional processing, I feel like I am mainly occupied with food: planning who wants to eat what and when, when to go shopping (also for the needy elderly with special preferences), unpacking the groceries from my bulk purchase, answering the question of what we’ll be eating, preparing meals, eating, clearing up and washing up ... it’s a bit like the Stone Age.”

In contrast to single parents, however, couples with children seem less worried about their financial situations. This is indicated by the fact that none of the topics identified by our topic model relates to materialistic worries or financial struggles.

While couples with children thusly also have childcare responsibilities, they did not face the same risk of loneliness than single parents did due to the (potential) presence of their partner (see Table 1). As a result, individuals who have both children and a partner tend to be somewhat less worried about social isolation. This is only true, of course, when the partners were actually physically and emotionally available.

### Living Alone

**FIGURE 5:**
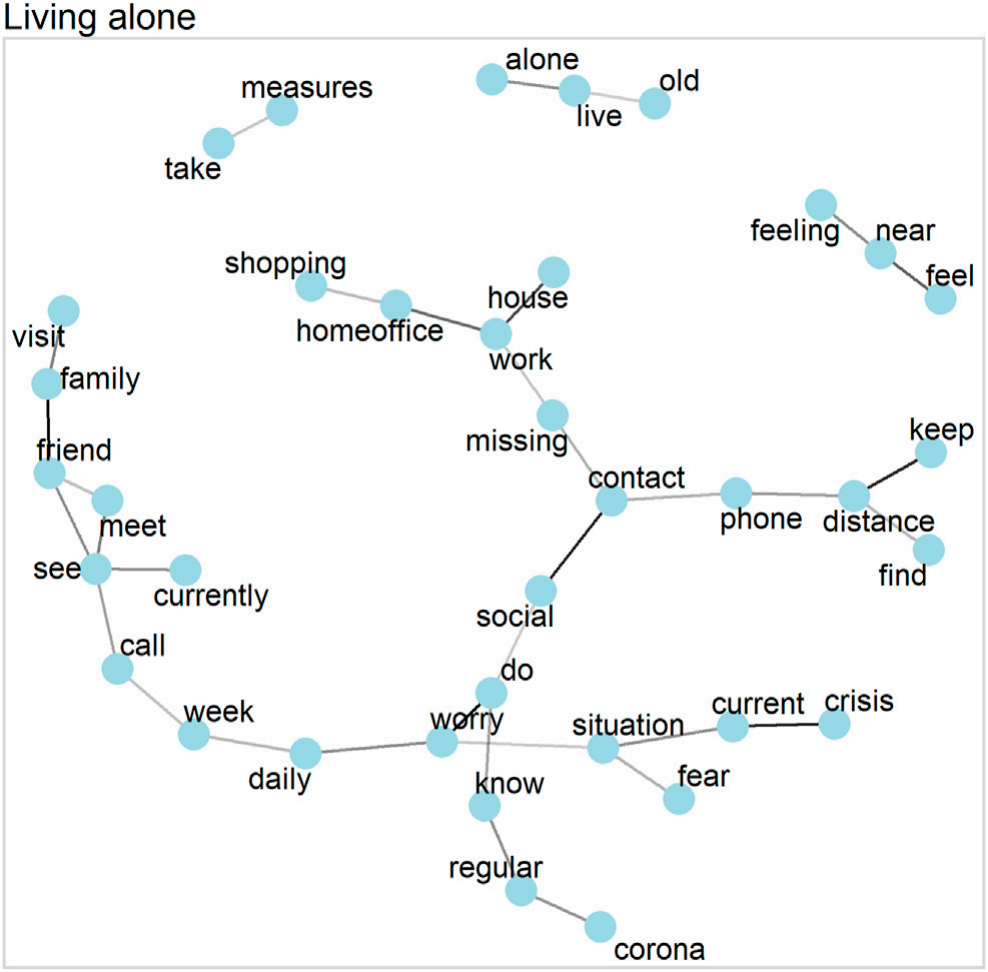
Word network for those living alone (correlations >0.25).

Respondents living alone have one big word network that deals primarily with social contacts (keeping distance, missing contacts, calling and/or visiting friends and/or family), as shown in Figure 5. This indicates the importance of social isolation and loneliness for this group.

**FIGURE 6:**
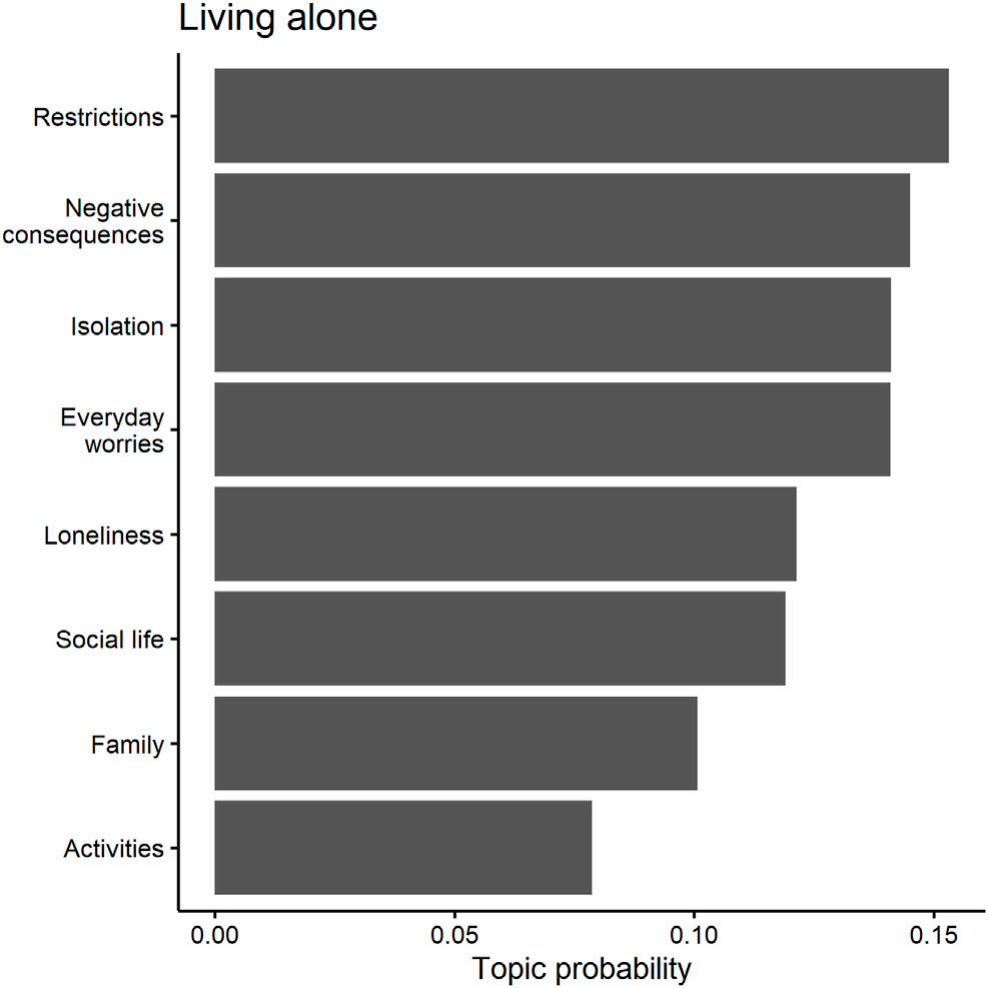
Predicted topic probabilities for those living alone.

The results of the topic models confirm that those living alone were mostly concerned about missing anything social. This is reflected by several topics that occur in this group (see Table 5). Examples are topics 5 and 6, which seem to relate to loneliness (topic 5: *missing*, *alone*) or social distancing (topic 6: *restrict*, *call*). Similarly, topic 7 relates to social life with terms such as *meeting* and *alone*. The three topics 5, 6 and 7 together make up about 42 percent of answers of those living alone, each occurring with about the same frequency (see Figure 6). Topics 5 and 6 are particularly relevant because they are what one would expect based on this group’s living situation. Noticeably, the answers that fall under these topics are mostly from the elderly. A retired woman over 60 reports her severe personal and psychological struggles from isolated living during the pandemic:

“My social contacts have been cut off due to choir and rehabilitation sport being cancelled. I have no contact with this group of people via e-mail, telephone or circulars, and I don’t dare to call anyone to just chat. Neighbors are becoming more important. Sometimes it seems to me that the ban on external contact has also led to an inner distancing. As a person with post-traumatic stress disorder, the situation reinforces my generalized anxiety disorder. Right now, I can’t see my therapist. I would like for mentally ill people to receive more care, such as a free therapy session by phone once a week. After [removed]’s suicide, I called the pastoral care by phone on Sunday to rediscover my balance. Right now, I don’t want to burden my friends with this.”

**TABLE 5:** Topics of those living alone (n = 199).

Topic 1	Topic 2	Topic 3	Topic 4	Topic 5	Topic 6	Topic 7	Topic 8
find	see	parents	daughter	missing	together	meet	week
restrict	find	fear	son	shopping	restrict	shopping	meet
corona	alone	economy	live	crisis	house	child	keep
job	fear	worry	house	phone	home office	alone	feel
week	shopping	crisis	do	alone	call	find	see
keep	worry	meet	worry	inside	difficult	see	most
home office	corona	hope	visit	due to	missing	financial	bad

Another woman over 60 reports similar concerns, but also points to the possibilities of online communication:

“I am sad that I cannot see my family (children, grandchildren) at Easter, which is what I miss most. On the other hand, I am glad that the Internet lets us see each other via regular video calls. I miss getting together with friends, sitting in the cafe in the sun.”

However, it is not just the elderly who face these problems. A man in his twenties reported:

“Personally, the current contact block is a major obstacle. I live alone and my partner lives further away. The crisis has limited how often we see each other, especially since we are dependent on the train and we want to use public transport as little as possible right now. I am also an active volunteer in a youth and sports club, but all work is currently suspended there. Currently, life mainly consists of working from home and the balance is missing, especially with a small apartment.”

A young student, who just started her bachelor’s degree, puts it rather bluntly:

“I’m super bored and I’d like to do something with friends, since it’s my first real semester break. Plus, I’m afraid I won’t be able to start my semester abroad in Australia, which is supposed to start in July.”

Those who live alone faced the highest risk of loneliness during lockdown conditions. At the same time, they did not have to take care of children (Table 1), which allowed them to dispose more freely of their time. We find that this combination can also be problematic, as the free time often cannot be used in a meaningful way. In the worst case, this can cause depressive symptoms or increase levels of anxiety.

### Shared Living Without Children

**FIGURE 7:**
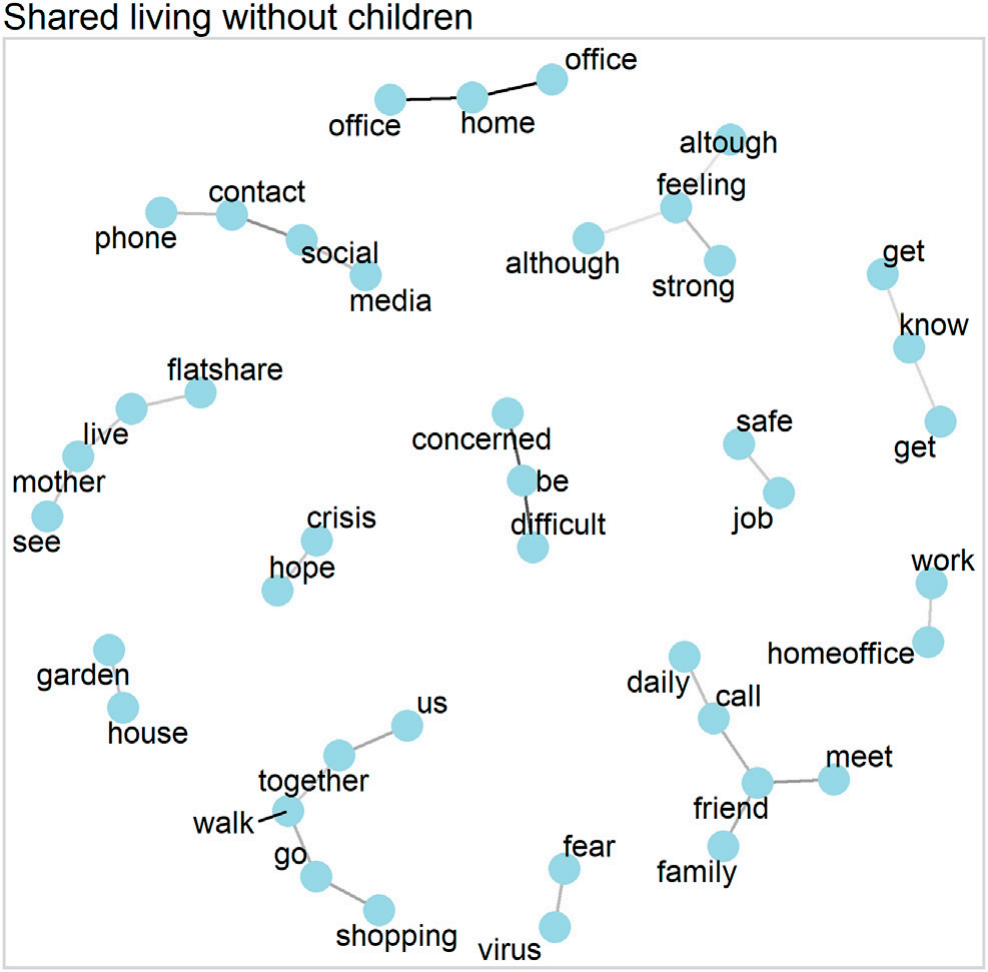
Word network for shared living without children (correlations >0.25).

Similarly to single parents, those in shared living situations without children also addressed several distinct issues, as Figure 7 shows. These issues include the use of online media and the telephone as a substitute for social contacts, or going out for shopping or a walk.

**FIGURE 8:**
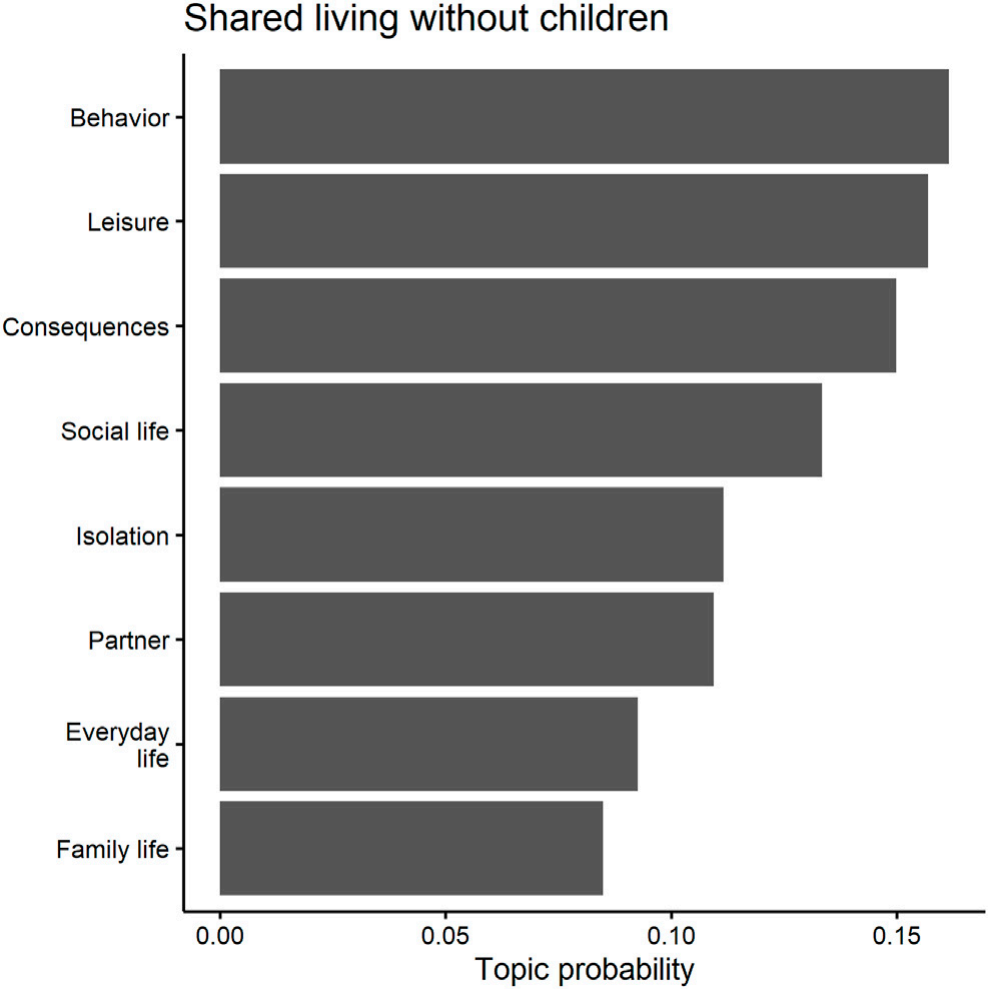
Predicted topic probabilities for shared living without children.

The topic models, shown in Table 6 and Figure 8, reveal that those who share a living space without children were most concerned about mundane issues such as missing sports or other free time activities (see Table 6). This is indicated by the dominance of topic 5, which contains leisure related words such as *sport* or *call*. With a probability of 18.1 percent, this topic stands out for those who share living without children (see Figure 8). It is the dominating topic of 93 of the 493 respondents who were in shared living conditions, but without children. The response of a woman in her twenties, who was living with her parents, exemplifies this well:

“Personally, I really miss my regular hobby appointments. I play an instrument in two orchestras and exercise twice a week. I also recently started lessons to get my motorcycle driver’s license together with my brother. I really miss the regular schedule of activities. And there is so much free time on weekends. This is completely unfamiliar. Of course, you’re happy when your weekend is completely free, but normally I am also happy to have something to do. I also miss getting together with my friends. Instead, we talk a lot on Skype. Still, I would like to go out to a restaurant, a bar, a cinema, a club etc. again. I really hope that this is over soon. Plus, it’s exhausting that there is nothing else to talk about.”

**TABLE 6:** Topics of those in shared living without children (n = 493).

Topic 1	Topic 2	Topic 3	Topic 4	Topic 5	Topic 6	Topic 7	Topic 8
worry	child	social	mother	week	crisis	house	restrict
social	shopping	current	house	miss	stop	partner	worry
changed	week	stop	shopping	sport	positive	strong	social
find	aware	corona	fear	home office	behave	corona	keep
positive	parents	find	daily	call	meet	worry	see
before	public	safe	out	house	parents	fear	home
together	space	burden	go	keep	heavy	meet	office

However, there might even be a positive side of the restrictions caused by COVID-19 for those without children yet not living alone, which is described by another woman of a similar age who seemed quite optimistic:

“Due to the initial restrictions (no more commuting for 2 h every day, no more evening activities) I have plenty of time at home, which I have wanted for years. Now I can have it without missing anything outside, since nothing is happening anyway. But I can pursue all of my hobbies without worries (student and secure job, which can be easily done from home): reading, meditating, writing, playing computer games, yoga, etc.”

While not all topics for those without children are positive, unsurprisingly, they do not address childcare-related problems. Moreover, financial worries also were less important in this group, with no topic occurring around such a theme.

Those who share their home with other people but who do not have childcare responsibilities should have the lowest risk of loneliness and, ceteris paribus, stress (see Table 1). Our results seem to be in line with this reasoning: respondents in this group were primarily concerned about issues related to leisure activities and worry significantly less about financial problems.

## Discussion and Conclusion

The central goal of this study was to illuminate how individuals living in different living arrangements experienced the first weeks of the COVID-19 pandemic in Germany. To that end, we used topic modeling on narratives collected through an open question in an online survey and content analysis. This is a topic of key relevance for social stratification research because it is well known that different types of living arrangements, such as single-parent or two-parent families, are associated with penalties and premiums, respectively, across several life outcomes. Yet, how living arrangements are associated with subjective experiences of the COVID-19 pandemic is still an under-researched topic. Therefore, this study contributes to the current academic debate on the relationship between household structure and resilience during the pandemic (Grasso et al., 2021).

Our results show that narratives differ substantially in the topics they cover and their variability, depending on the type of living arrangement. Those who live alone and couples with children seem to focus on a few dominant topics (i.e., isolation and childcare), while single parents report on a broad spectrum of topics with a particular focus on the financial situation and childcare. Adults living with others but without children are most likely to write about leisure activities and report positive as well as negative experiences. Hence, individuals in shared living arrangements without children seem to cope best with the crisis and tend to report concerns that could be regarded as more trivial. Parents especially seem to struggle during the social restriction periods due to issues related to childcare, finances, and the absence of a social support network. Finally, individuals living alone are mostly concerned with their social isolation. While this topic is present in all four groups, it is most salient for the group of single households.

Our study highlights that individual experiences varied greatly during the first weeks of the COVID-19 pandemic in Germany depending on individual living arrangements and how the families of respondents were structured. In line with our expectations, depending on their living arrangement, individuals are unequally susceptible to the negative social consequences of the pandemic. While those living alone were mostly at risk of suffering from social isolation, parents faced issues related to childcare and finances, which is most pronounced for single parents, who are usually women. In contrast, those in shared living accommodations without kids were mostly concerned with changes in their leisure time and were coping with the crisis better than the other analyzed groups. These results suggest that cohabitation can increase peoples’ resilience and wellbeing compared to individuals who live alone. However, the lack of childcare facilities and social support networks can cause severe issues for parents during the crisis, especially for single parents. On a practical level, our results imply that social policies should be specifically designed to help vulnerable groups with their specific needs.

While we are confident that our study provides valuable insights, the results have some limitations. These point towards potential avenues for methodological refinement and further scholarly inquiry. First, as the sample is not representative of the entire German population and it oversamples young, highly educated respondents, we expect that financial struggles and health concerns are considerably stronger among the elderly and less educated. However, we believe this is a first important step in research analyzing the relationship between living arrangement structure and experiences of the pandemic. Future studies might want to replicate our findings using nationally representative data and observe whether our conclusions change. Likewise, while studies reported lower levels of wellbeing of gender minorities during the pandemic (Buspavanich et al., 2021), studies concerned with the subjective experience of non-heterosexual fathers and mothers indicate that these individuals experienced their childcare responsibilities during the pandemic more positively (Craig and Churchill, 2021). Hence, there could be an interaction effect between household type and gender minority status for experiencing the pandemic.

Given that our sample is slightly biased towards upper-class, younger individuals, we expect results coming from representative data to find even stronger associations that those reported in this study. This assumption is in line with representative data gathered during the first weeks of the pandemic in Germany (Naumann et al., 2020). Second, in the household category of “shared living without children,” we are unable to distinguish between those individuals that were sharing a house with flatmates (e.g., university students), those living in inter-generational households (e.g., grandparents and a grandchild) or those who are couples, cohabiting or married, without children. This limitation means that in this category we have a mix of living arrangements. However, even though we are not strictly comparing concerns across all possible types of arrangements, we are confident that our results highlight important insights on how different types of household structures might compensate for the negative effects of the pandemic. Future studies should carefully distinguish types of households, living arrangements and family structures, and possibly observe how coping mechanisms and experiences vary across these dimensions. Third, we have only captured impressions of the early stages of the pandemic in Germany. Therefore, we cannot draw any conclusions about whether the results will change over the course of the pandemic. We speculate, however, that the critical differences by living arrangements we found in our study are likely to be even stronger the longer the pandemic-related restrictions are in place. Our fieldwork was conducted in Germany when the country was among those affected the least by COVID-19 in terms of both infections and restrictions. By the end of 2020, however, Germany was characterized by rising infection rates and, although not with a lockdown of the strictest type, regulations were severely constraining every-day life and work for long periods. The evolution of such a crisis calls for new research examining the long-term consequences of the pandemic by household type. Furthermore, we only differentiate between traditional household structures (i.e., single households, single parents, families with children and shared living without children), which might ignore the growing diversity of families and household structures, like same-sex couples, for instance. Moreover, an international perspective on how concerns vary between welfare states is a promising field of investigation. Different contexts provide different resources for households and families. For example, while shared physical custody is the norm in Scandinavia, this represents a rare resource in our case study, Germany. Such differences are key to understanding variations in concerns *within* a household type (i.e., single-parent families) *between* countries. Therefore, we expect some welfare states to provide greater compensatory resources for those most concerned, like single-parent families. Ultimately, this would lead to the hypothesis that in socio-democratic welfare states differences in concerns by living arrangement will be *smaller* than the differences found in a conservative-type welfare state like Germany.

While we acknowledge these limitations, we are confident that our study provides important insights into the research question at hand. The primary goal of our study lied in describing how individuals felt during the beginning of the COVID-19 restrictions in Germany, differentiated by an important characteristic: their personal living arrangements. This does not imply that living arrangements are the single cause of a certain phenomenon, such as loneliness or financial hardship. Estimating such causal effects is usually limited to analyzing single, closed survey items. In contrast, we analyze detailed, open answers of respondents that do not force people into a one-dimensional and pre-defined scale. Future studies might want to dig even deeper into how narratives of the pandemic vary across households. Using techniques like in-depth interviews or focus groups should bring additional insights into how different household configurations are associated with discourses, perceptions and possible strategies to cope with the consequences of the pandemic. However, such qualitative studies would unlikely be able to cover the large number and thereby variety of individuals our data encompass.

In the broader picture, our study adds to the growing body of studies that highlight how the COVID-19 pandemic fosters social inequality. In light of the issues that some respondents reported with childcare, our study confirms that potential gender inequalities need special attention (Kreyenfeld et al., 2020; Möhring et al., 2020). This concerns single parents, a group predominantly consisting of mothers. Furthermore, our study provides insights into the issue of social isolation and loneliness. Given that initial empirical evidence confirms that loneliness has surged during the pandemic, as have the manifold mental health issues associated with loneliness, our study offers multiple insights into which living conditions prevent or foster loneliness during social restrictions (Killgore et al., 2020).

The narrative analysis provides empirical evidence that private living arrangements are an important factor in how respondents cope with the crisis and what central concerns they experience. Given that the pandemic seems to burden already vulnerable social groups (i.e., the mostly female single parents), our study provides additional evidence that social policies have to support these social groups in need even more.

## Data Availability

The datasets presented in this study can be found in online repositories. The names of the repository/repositories and accession number(s) can be found below: https://data.gesis.org/sharing/#!Detail/10.7802/2034.

## Footnotes

1 For more information on the sample and data collection, see: https://doi.org/10.7802/2034.

2 Note that the total numbers differ by a few cases from the *n* of the variables in Table 2. This is because some variables that are not part of our statistical model (e.g., education or age) have missing values for a few respondents. To draw upon as much information as possible, we decided against listwise deletion for those variables we show to describe our sample.

3 All translations were done with the free version of the neural machine translation service DeepL: www.DeepL.com/Translator, with manual corrections in some cases to improve readability and to remove information that could potentially reveal identities from the quoted answers.
